# Characterization of Two Second-Site Mutations Preventing Wild Type Protein Aggregation Caused by a Dominant Negative *PMA1* Mutant

**DOI:** 10.1371/journal.pone.0067080

**Published:** 2013-06-25

**Authors:** Pilar Eraso, Francisco Portillo, María J. Mazón

**Affiliations:** 1 Departamento de Bioquímica, Consejo Superior de Investigaciones Científicas, Universidad Autónoma de Madrid, Madrid, Spain; 2 Instituto de Investigaciones Biomédicas “Alberto Sols”, Consejo Superior de Investigaciones Científicas, Universidad Autónoma de Madrid, Madrid, Spain; Simon Fraser University, Canada

## Abstract

The correct biogenesis and localization of Pma1 at the plasma membrane is essential for yeast growth. A subset of *PMA1* mutations behave as dominant negative because they produce aberrantly folded proteins that form protein aggregates, which in turn provoke the aggregation of the wild type protein. One approach to understand this dominant negative effect is to identify second-site mutations able to suppress the dominant lethal phenotype caused by those mutant alleles. We isolated and characterized two intragenic second-site suppressors of the *PMA1*-D378T dominant negative mutation. We present here the analysis of these new mutations that are located along the amino-terminal half of the protein and include a missense mutation, L151F, and an in-frame 12bp deletion that eliminates four residues from Cys409 to Ala412. The results show that the suppressor mutations disrupt the interaction between the mutant and wild type enzymes, and this enables the wild type Pma1 to reach the plasma membrane.

## Introduction

The proton pumping H^+^- ATPase of *Saccharomyces cerevisiae*, Pma1 [1], is an electrogenic P-type cation pump [2] that is essential for the uptake of nutrients and the regulation of intracellular pH [3]. It is well established by homology modeling based on X-ray diffraction studies of different ATPases that sequence homology in P-type ATPases family is restricted to discrete regions but that the overall structure is conserved even among different subfamilies [4]. Pma1 is an abundant integral membrane protein destined to the cell surface where it spans the lipid bilayer with 10 transmembrane segments [5]. The cytoplasmic part of the protein contains three functional domains: the nucleotide-binding domain (N-domain), the phosphorylation domain (P-domain), and the actuator domain (A-domain) [6]. In addition, Pma1 as well as other P-type ATPases contains a C-terminal regulatory domain [7]. Due to its abundance Pma1 constitutes a major cargo of the secretory pathway and its amount in the plasma membrane is tightly controlled [8]. In fact *PMA1*overexpression induces cell stress and, as a consequence, the activation of the Hog1-MAPK pathway [9].

Pma1 is biosynthetically inserted into the endoplasmic reticulum (ER) membrane and after folding and homo-oligomerization travels to its final destination through a series of transport vesicles [10], [11], [12]. In its way to the membrane it becomes associated with lipid rafts, a process that occurs in the ER [13]. Correct oligomerization of Pma1, as well as its stability in the plasma membrane, requires long-chain fatty acid containing lipids [12], [14], [15], [16], but the Pma1 regions involved in the formation of oligomers remain to be determined. Mutant Pma1 proteins that are not properly folded do not reach the plasma membrane but rather remain accumulated in ER-derived structures that stain with anti Kar2 antibodies [17].

*PMA1*, the gene coding for the plasma membrane H^+^-ATPase, is an essential gene that has been extensively studied by site-directed mutagenesis [18]. Among the large number of mutants generated several point mutations have been shown to cause cell growth arrest even in the presence of a co-expressed wild type *PMA1* allele. Particularly, mutations altering Asp378, a residue conserved in all P-type ATPases, exhibit this dominant lethal phenotype [17], [19], [20], [21]. The Asp378 residue is located in the central cytoplasmic loop of the protein and is involved in the formation of the aspartyl-phosphate intermediate during the catalytic cycle of the enzyme. *PMA1* alleles carrying changes in this residue produce misfolded proteins that are retained in the ER [17], [20], [21] and finally degraded by the Endoplasmic Reticulum Associated Degradation (ERAD) machinery [22], [23]. The efficient degradation of the mutant Pma1 variants requires in addition an intact autophagy pathway [23]. The ERAD mechanism involves the recognition of the misfolded proteins in the ER, their ubiquitination and retrotranslocation into the cytoplasm, and the degradation of the proteins by the proteasome [24], [25], [26], [27], One example of these dominant lethal alleles is *PMA1*-D378T whose expression not only produces a misfolded protein [22], [28], but also interferes with the folding of the wild type protein leading to the aggregation of both mutant and wild type proteins, and their slow degradation by ERAD [28]. As a consequence, Pma1 protein is depleted from the cell surface causing growth arrest. In an attempt to understand the mechanism of formation of protein aggregates and how dominant lethal Pma1 affects the wild type enzyme we have isolated intragenic revertants of dominant Pma1 mutants. We have previously described a suppressor mutation for *PMA1*-D378T dominant lethality, Trp859stop, which disrupts the formation of oligomers between the mutant and wild type enzymes [28]. The Trp859stop suppressor mutation deletes transmembrane segment 10 (TM10) and the C-terminus of the Pma1 protein suggesting the involvement of this transmembrane segment in the oligomerization of the enzyme. Here, we describe and characterize two new suppressor mutations for *PMA1*-D378T mutant that localize to TM2 (L151F) and the N-domain (ΔC409-A412).

## Materials and Methods

### Yeast Strains and Growth Conditions

Yeast strain BY4741 (*MAT*a *his*3Δ1 *leu2*Δ0 *met15*Δ0 *ura3*Δ0) purchased from EUROSCARF (Frankfurt, Germany) was used in all the experiments. Synthetic media containing 0.67% Yeast Nitrogen Base w/o amino acids, 2% dextrose (SD), 2% raffinose (SR) or 2% galactose (SG) and the appropriate requirements were used. Standard methods for yeast transformation, culture and manipulation were used [29]. For growth tests the different yeast strains were grown on SR for 24 h, suspended in water to an OD_660_ = 0.1 and 5 µl were dropped on SD and SG agar plates.

In cycloheximide-chase experiments the different transformants were grown in SR medium to mid-exponential phase (OD_660_ = 0.8–1), galactose was added to the culture at a final concentration of 2% and cells were cultured during 4 h to induce the expression of the *PMA1* alleles. Cycloheximide was added to a final concentration of 200 µg/ml and samples of 50 OD were taken at the indicated times and frozen in liquid nitrogen.

### Plasmids

Plasmids pIB2010, pIB2018, pIB2019 and pIB2039 are derivatives of the *LEU2* centromeric plasmid pRS315 [30] and carry myc-tagged *PMA1*-D378T (pIB2010) or *PMA1* (pIB2018) and HA-tagged *PMA1*-D378T (pIB2019) or *PMA1* (pIB2039) alleles under the control of the *GAL1* promoter. The construction of these plasmids has been reported [23], [28]. Site-directed mutagenesis was used to introduce the L151F and ΔC409-A412 mutations in a 4.3 kb *Xho*I-*Hind*III fragment containing the HA-tagged *PMA1* gene subcloned into pSK vector (Stratagene, La Jolla, CA) using the QuickChange™ method developed by Stratagene and modified as in [31]. The correct introduction of the mutations was verified by sequencing. Plasmids carrying the single mutants HA-*pma1*-L151F and HA-*pma1*-ΔC409-A412 under the control of the *GAL1* promoter were generated by cloning a 0.7 kb *EcoR*I-*Xho*I fragment containing the entire *GAL1* promoter [32] and the 4.3 kb *Xho*I-*Hind*III fragment containing the corresponding mutagenized HA-tagged *PMA1* allele into the *EcoR*I and *Hind*III sites of the *URA3* single copy plasmid pRS316 [30].

### Isolation and Sequencing of Revertants from*PMA1*-D378T

Wild type yeast strain BY4741 was transformed with 50 µg of pIB2010 using the lithium acetate procedure [33]. Approximately 12,000 transformants were selected in SR medium. Transformed cells were replica plated onto SG medium to induce the expression of the dominant lethal allele. Plasmids were rescued [29] from those yeasts able to grow on galactose medium and retransformed to confirm their ability to suppress the dominant lethal phenotype of the D378T mutant ATPase. Inserts contained in the suppressor plasmids were sequenced.

### Cell Lysis and Immunoblotting

Total yeast protein extracts were prepared by vortexing with glass beads in buffer containing 50 mM Tris-HCl pH 8, 5 mM EDTA and a protease inhibitor cocktail (Roche, Manheim, Germany). The homogenate was cleared from intact cells by centrifugation for 10 min at 300×g. The supernatant was then centrifuged at 15,000×g for 20 min. The resulting pellet was resuspended in buffer containing 20% w/v glycerol, 50 mM Tris-HCl pH 8, 5 mM EDTA, 2 mM DTE and protease inhibitor cocktail. Proteins were resolved in 8% SDS-polyacrylamide gel electrophoresis [34] and transferred to a PVDF membrane (Immobilon P, Millipore) for immunodetection using mouse monoclonal 12CA5 (Roche, Manheim, Germany) against the HA epitope, rabbit polyclonal antibody against the myc epitope (Sigma, St Louis, MO), rabbit polyclonal anti-Kar2 (Santa Cruz Biotechnology) or mouse monoclonal anti-Pma1 [35]. Immunodetection was performed using the ECL Western blot analysis system (Amersham Biosciences, Sunnyvale, CA) according to the manufacturer’s protocol. Protein concentration was determined by the Bradford method [36] with the Bio-Rad Protein Assay reagent (Bio-Rad, Hercules, CA) and bovine IgG as standard.

### Immunofluorescence

Yeast cells coexpressing HA-tagged and myc-tagged *PMA1* alleles were cultured in SR medium overnight at 30°C, and galactose was added to the culture at a final concentration of 2% for 4 h at 30°C to induce the expression of *GAL1*-dependent genes. Yeast cells were fixed and stained for immunofluorescence as described [37], using mouse monoclonal 12CA5 anti-HA and rabbit polyclonal anti-myc antibodies as primary antibodies, followed by FITC-conjugated goat anti-mouse or rhodamine-conjugated goat anti-rabbit (Jackson ImmunoResearch, West Grove, PA). Images were obtained with a Zeiss Axiofot microscope and further processed with Adobe Photoshop 7.0.

### Blue Native Gel Electrophoresis

Solubilization of membrane proteins for blue native gel electrophoresis was performed using a modification of the method described in [38]. Twenty µg of protein were solubilized in 20 µl of buffer containing 50 mM Tris-HCl pH 7.4, 5 mM EDTA, 750 mM 6-aminocaproic acid, 1 mM PMSF and either 3% Triton X-100 or 1% SDS. Samples were incubated at 4°C in the case of Triton X-100 solubilization, or at room temperature in the case of SDS, for 30 minutes and centrifuged for 5 min at 15,000×g. Proteins in the supernatant were separated on 4–16% polyacrylamide gradient gels using NativePAGE Novex Bis-Tris System with NativeMark Unstained Protein Standard (Invitrogen, Carlsbad, CA). NativePAGE Sample Buffer was supplemented with 750 mM 6-aminocaproic acid and 0.75% G-250 Sample Additive. Proteins were transferred to PVDF and analyzed by immunoblotting.

## Results

### Isolation of Suppressors of the Dominant Lethal Phenotype Caused by*PMA1*-D378T

Revertants of the dominant lethal phenotype caused by the *PMA1*-D378T mutation were obtained as described in Materials and Methods. Briefly, wild type cells carrying the dominant lethal *PMA1*-D378T allele under the control of the *GAL1* promoter were grown in raffinose medium and replica plated to galactose-containing plates. From 12,000 colonies analyzed, 10 were able to grow on galactose. Plasmid DNA was isolated from those colonies to determine if they were full revertants or either intra- or extragenic suppressors. Upon retransformation and sequencing it was found that 3 of the revertants were due to second-site mutations in the *PMA1*-D378T mutant allele while the rest were reversions to wild type.

**Figure 1:**
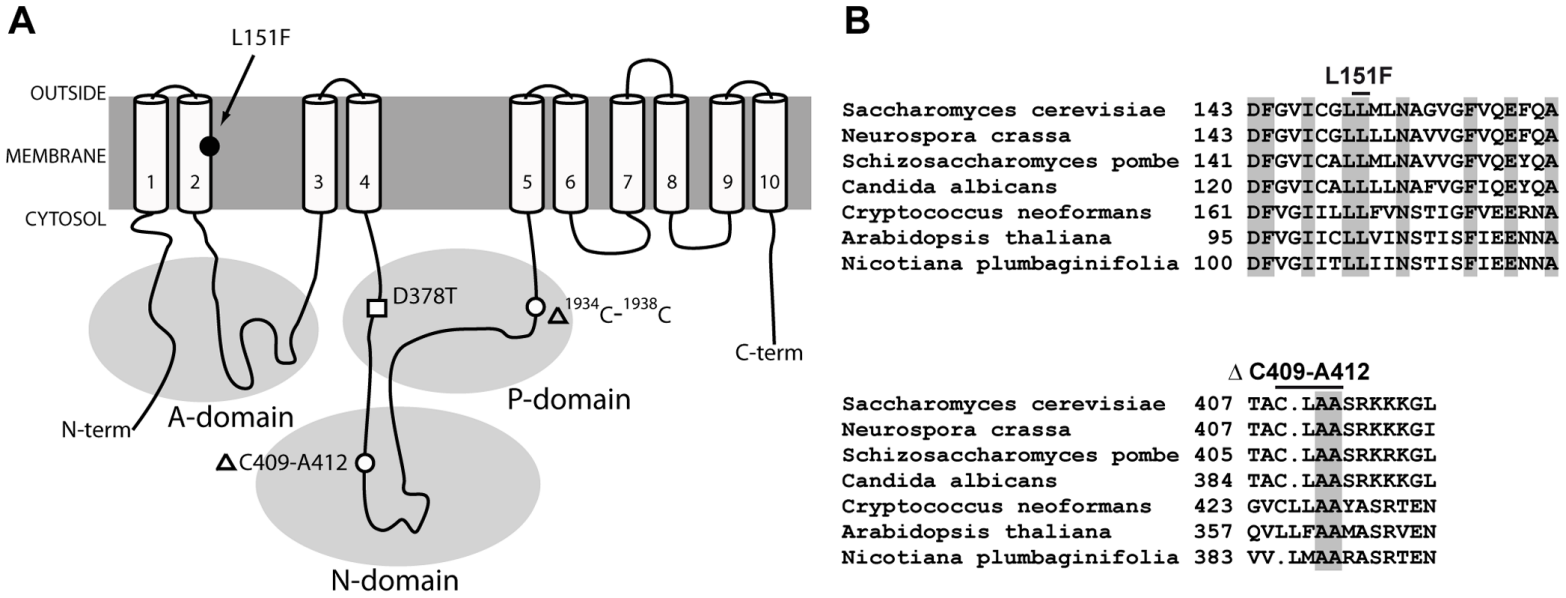
Mutations that suppress the dominant lethal phenotype of*PMA1*-D378T allele. **A)** Location of the suppressor mutations found in the revertants. The positions where suppressor mutations create a missense mutation (closed circle) or deletions (open circles) are shown on a topological model of the ATPase. The position of the original mutation *D378T* (open square) is also shown. The scheme shows the proposed functional domains of the Pma1 protein as extrapolated from the crystal structure of the *Arabidopsis thaliana* homologous ATPase: a transmembrane domain with ten helices and three cytosolic domains named A (actuator domain), P (phosphorylation domain) and N (nucleotide-binding domain). **B)** Alignment of the sequences of several H^+^-ATPase family members in the region surrounding mutations L151F and ΔC409-A412.

**Figure 2:**
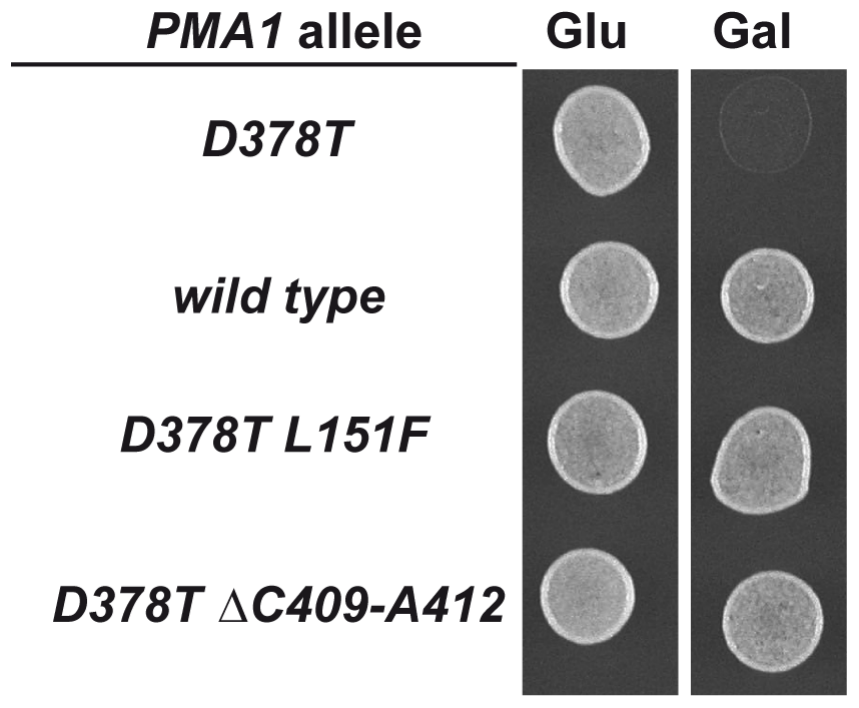
Growth of cells carrying wild type and mutant Pma1 variants. Drop test for growth on galactose of yeast strains carrying the indicated *PMA1* alleles, all of them under the control of the *GAL1* promoter. The different strains were grown in SR medium for 24 h, suspended in water to an OD_660_ = 0.1 and 5 µl were dropped on SD and SG agar plates. Identical results were obtained with three independent transformants.

One of the suppressor mutations found is a deletion of 25 base pairs, from ^1934^C to ^1958^C, in the P-domain of the Pma1 protein. The deletion produces a frame-shift that creates a stop codon and leads to a truncated protein with an apparent molecular weight of 80 kDa. This frameshift mutation, renders a Pma1 variant lacking six transmembrane segments, TM5 to TM10, and the carboxy-terminal regulatory domain. Several C-terminal truncations of Pma1 have been constructed and analyzed in the past [27], [39], with respect to their trafficking, stability and degradation. For this reason we did not pursue any further the characterization of this suppressor. Another suppressor mutation found is a missense mutation that causes a leucine to phenylalanine change at amino acid 151 (L151F). Finally, a third suppressor mutation is a deletion of 12 base pairs that leads to loss of four residues, Cys409, Leu410, Ala411 and Ala412. Figure 1A depicts the location of the suppressor mutations. The missense mutation L151F is located within transmembrane segment TM2. Genetic evidence suggests that the first and second transmembrane segments of Pma1 form a helical hairpin with important structural determinants that contribute to its overall flexibility [40]. The ΔC409-A412 deletion, located in the N-domain, starts 31 residues downstream of Asp378 where the formation of the catalytic intermediate takes place. Biochemical and genetic evidences suggest that Cys409 is an important determinant in the local structure surrounding the phosphorylation site [41]. Sequence comparison (Figure 1B) shows that Leu151 is conserved in all the H+-ATPases. Among the amino acids eliminated by the ΔC409-A412 deletion, Ala411 and Ala412 are also conserved in all the H^+^-ATPases while conservation of Cys409 and Leu410 is restricted to fungal H^+^-ATPases. In Figure 2 the growth of wild type cells transformed with the plasmids harboring the intragenic suppressors *pma1*-D378T/L151F and *pma1*-D378T/ΔC409-A412 shows how the overexpression of these new alleles allows growth on galactose-containing medium.

As mentioned above not only mutations altering the Asp378 residue but also other point mutations in the *PMA1* gene exhibit a dominant lethal phenotype [19]. We then asked whether the suppressor mutations found here are able to suppress other dominant lethal mutations or are specific of D378T. To answer this question we combined in *cis* the L151F and ΔC409-A412 suppressor mutations with several dominant lethal mutations, namely D378E, K474R, D560N, D634N and R695L. Dominant lethality was suppressed in all cases indicating that the suppressor mutations L151F and ΔC409-A412 are not specific for the D378T allele. The suppression of mutations D560N and R695L is shown as an example (Figure S1).

### Characterization of the Suppressors

**Figure 3:**
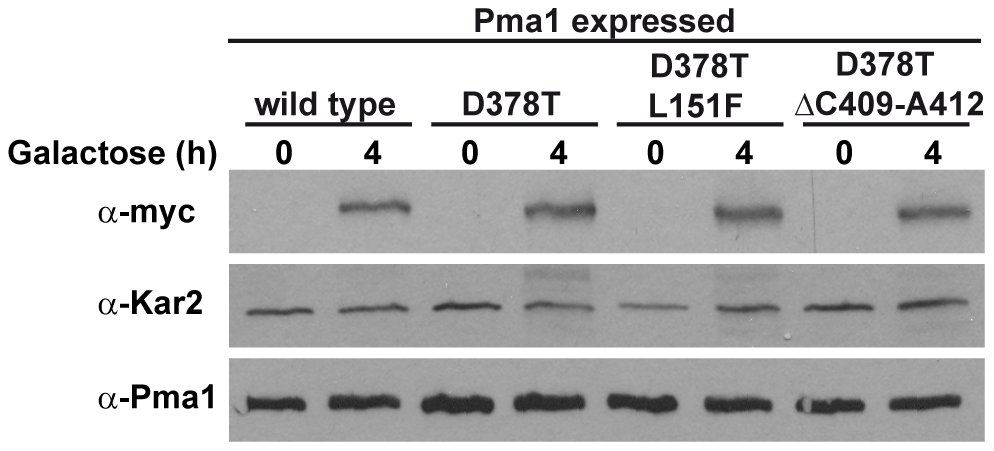
GAL1-induced expression of wild type and mutant Pma1 variants. Yeast strains carrying the indicated myc-tagged *PMA1* alleles, all of them under the control of the *GAL1* promoter, were grown in SR medium and derepressed in galactose containing medium for 4 h to induce the expression of the *PMA1* alleles. Samples were taken at times zero and 4 h of induction and total yeast membranes were prepared and analyzed by Western blot with anti-myc and anti-Kar2 antibodies. The same samples were loaded in a duplicate gel and immunoblotted with monoclonal anti-Pma1 antibody.

**Figure 4:**
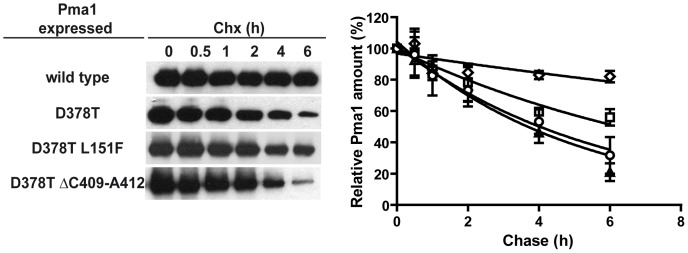
Stability of the wild type and mutant Pma1 variants. Yeast strains carrying the indicated myc-tagged *PMA1* alleles under the control of the *GAL1* promoter, were grown in SR medium and derepressed in galactose containing medium for 4 h to induce the expression of the different Pma1 proteins. After addition of cycloheximide, samples were taken at the indicated times and total yeast membranes prepared and analyzed by Western blot with anti-myc antibodies. A representative blot of each cycloheximide-chase experiment is shown. Densitometric analysis of the immunoblots was performed and remaining Pma1 at different times was referred to the amount before adding cycloheximide (t = 0). Wild type (diamonds), Pma1-D378T (circles), Pma1-D378T/L151F (squares), Pma1-D378T/ΔC409-A412 (triangles). Shown are the means and standard deviations of three independent experiments calculated with GraphPad Prism5.

Dominant lethal *PMA1* alleles render misfolded proteins that are retained in the ER [17], [20], and targeted for degradation by ERAD [22], [27], [23]. To characterize the mechanism of suppression of the new mutations we first analyzed whether the revertant enzymes containing the D378T/L151F and D378T/ΔC409-A412 mutations could exhibit a reduced expression in galactose medium as compared with that of the dominant lethal Pma1-D378T protein. We compared the extent of induction during 4 h in galactose-containing medium of the *GAL1*-driven *PMA1* alleles: wild type, D378T, D378T/L151F and D378T/ΔC409-A412. As can be seen in Figure 3 the Pma1 variants encoded by these alleles are induced to a similar extent when the cells are shifted to galactose. This result indicates that the suppression observed cannot be explained by low or lack of induction of the revertant proteins. Next we explored the possibility that the revertant enzymes could exhibit an increased degradation rate as compared with the Pma1-D378T mutant protein. For this purpose we performed cycloheximide-chase analysis in wild type cells expressing each of the mentioned alleles. After growth in raffinose medium the expression of the mutant proteins was induced with galactose for 4 hour and the amount of remaining myc-tagged Pma1 variants after addition of cycloheximide was examined by western blot along 6 hour. Figure 4 shows that the stabilities of Pma1-D378T/L151F and Pma1-D378T/ΔC409-A412 proteins showed no significant difference with that of Pma1-D378T. The stability of the mutant proteins was also determined in cells lacking Ubc7, an ubiquitin conjugating enzyme, required for ERAD of Pma1 [23], in order to assess the dependence of their degradation on ERAD. The stabilization observed for the mutant proteins in the *ubc7* background (Figure S2) suggests the involvement of the ERAD mechanism.

**Figure 5:**
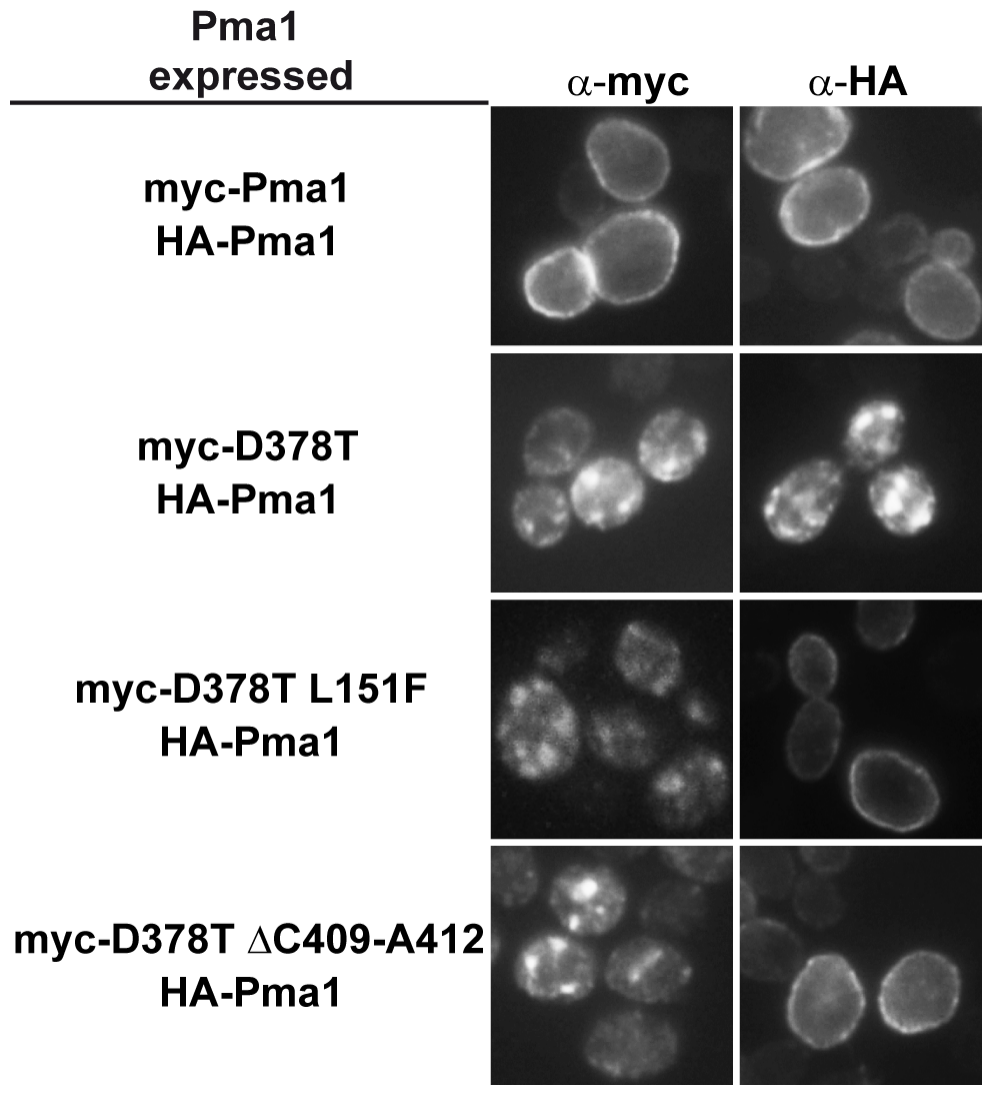
Localization of wild type and mutant proteins. Yeast strains expressing *GAL1*-driven HA-tagged wild type Pma1 and *GAL1*-driven myc-tagged wild type Pma1, dominant lethal Pma1-D378T, or double mutant proteins Pma1-D378T/L151F and Pma1-D378T/ΔC409-A412 were processed for indirect immunofluorescence microscopy as described in Materials and Methods. Monoclonal anti-HA and FITC-conjugated secondary antibodies were used to detect HA-Pma1 while polyclonal anti-myc and rhodamine-conjugated secondary antibodies were used to detect the myc-tagged Pma1variants.

We next analyzed the subcellular localization of the mutant proteins by indirect immunofluorescence. Wild type cells were co-transformed with plasmids containing HA-tagged wild type *PMA1* and each of the myc-tagged double mutant *PMA1* alleles. Cells co-transformed with the wild type and dominant lethal *PMA1*-D378T alleles were used as control. Expression of dominant *PMA1* alleles has been extensively shown to lead to the formation of intracellular ER-derived punctate structures that contain the mutant protein and the co-expressed wild type protein [17], [20], [42], [28]. Cells were grown in raffinose and shifted to galactose-containing medium to allow expression of the *GAL1*-driven *PMA1* alleles contained in the plasmids. The location of the HA- and myc-tagged proteins was analyzed after 4 h induction. As can be seen in Figure 5, in cells expressing the alleles containing both mutations, D378T and one of the suppressor mutations, the co-expressed HA-tagged Pma1 localized to the plasma membrane whereas the myc-tagged double mutant proteins remained in intracellular punctate structures. In control cells, containing both HA-Pma1 and myc-Pma1 the antibodies decorated the plasma membrane, as expected, whereas when HA-Pma1 and myc-Pma1-D378T were co-expressed the anti-HA and anti-myc antibodies decorated the punctate structures as described. These results suggest that the presence of the suppressor mutations abolishes the co-retention of wild type Pma1 and permits its normal traffic to the plasma membrane allowing cell growth. None of the two suppressor mutations however allow the mutant proteins to escape, not even partially, from the punctate structures.

### Characterization of the Single Mutant Proteins

**Figure 6:**
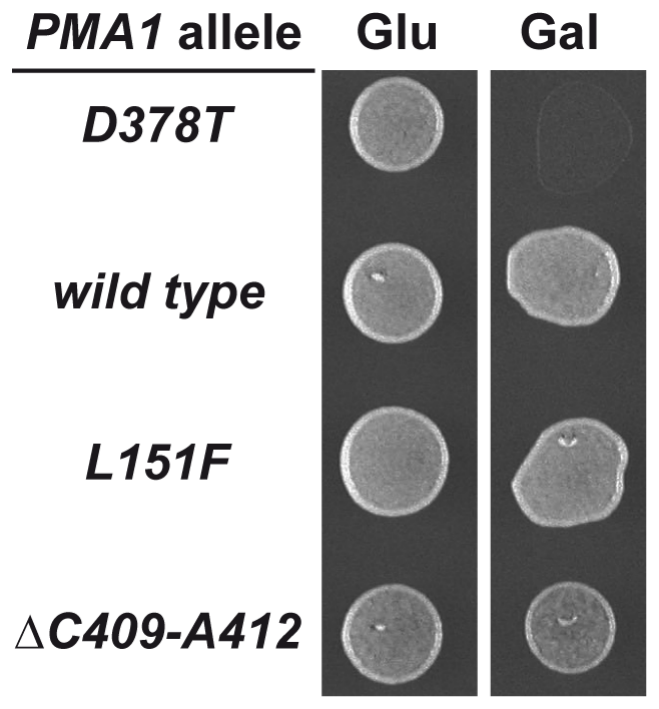
Growth of cells carrying wild type and single mutant Pma1 variants. Drop test for growth on galactose of yeast strains carrying the indicated *PMA1* alleles, all of them under the control of the *GAL1* promoter was performed as in Figure 2. Identical results were obtained with three independent transformants.

**Figure 7:**
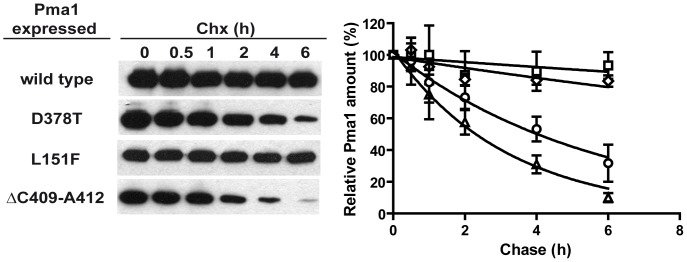
Stability of the wild type and single mutant Pma1 variants. Yeast strains carrying the indicated HA-tagged *PMA1* alleles under the control of the *GAL1* promoter, were grown in SR medium and derepressed in galactose containing medium for 4 h to induce the expression of the different Pma1 proteins. Samples were taken and processed as indicated in Figure 4 and analyzed by Western blot with anti-HA antibodies. A representative blot of each cycloheximide-chase experiment is shown. Densitometric analysis of the immunoblots was performed and remaining Pma1 at different times was referred to the amount before adding cycloheximide (t = 0). Wild type (diamonds), Pma1-D378T (circles), Pma1-L151F (squares), Pma1-ΔC409-A412 (triangles). Shown are the means and standard deviations of three independent experiments calculated with GraphPad Prism5.

**Figure 8:**
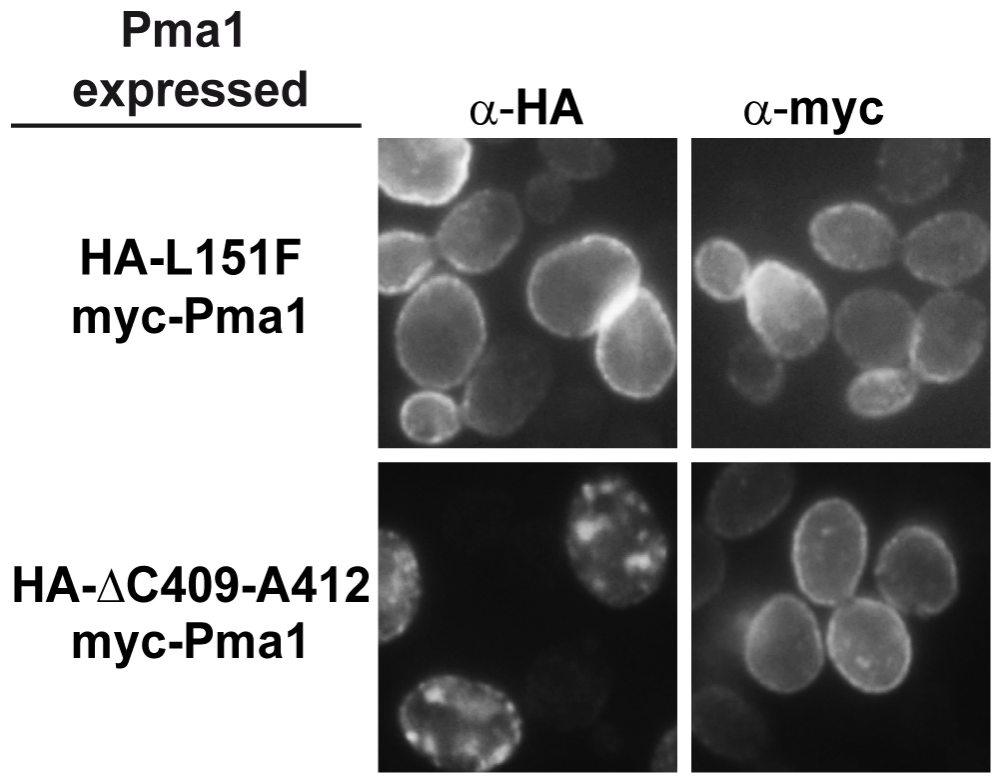
Localization of wild type and single mutant proteins. Yeast strains expressing *GAL1*-driven myc-tagged wild type Pma1 and *GAL1*-driven HA-tagged single mutant proteins Pma1-L151F or Pma1-ΔC409-A412, were processed for indirect immunofluorescence microscopy as described in Materials and Methods. Monoclonal anti-HA and FITC-conjugated secondary antibodies were used to detect HA-Pma1 while polyclonal anti-myc and rhodamine-conjugated secondary antibodies were used to detect the myc-tagged Pma1variants.

To learn more about the impact of the second-site mutations on the Pma1 protein we constructed *PMA1* alleles containing only the suppressor mutations and studied the degradation rate and subcellular localization exhibited by the single mutant proteins, Pma1-L151F and Pma1-ΔC409-A412. The single mutant alleles were constructed by site-directed mutagenesis, tagged with the HA epitope at the amino terminus, and cloned under the control of the *GAL1* promoter in a centromeric plasmid. Growth of cells carrying the single mutant *PMA1* alleles can be seen in Figure 6. Half-life of the mutant proteins was determined by cycloheximide pulse-chase experiments after 4 h induction in galactose-containing medium. The degradation rate of the Pma1-L151F protein was comparable to that of the wild type protein. In the case of Pma1-ΔC409-A412 its degradation rate was accelerated as compared with that of the mutant Pma1-D378T (Figure 7). When the subcellular localization of the mutant proteins was analyzed by indirect immunofluorescence we observed that while in cells expressing HA-Pma1-L151F the anti-HA antibody stained the plasma membrane, HA-Pma1-ΔC409-A412 mutant protein remained in intracellular punctate structures (Figure 8). These results indicate that cells expressing Pma1-L151F mutant protein behave as wild type with respect to growth. Also, the stability and traffic to the plasma membrane of the isolated L151F mutant are similar to those of the wild type protein, while the variants carrying the ΔC409-A412 deletion, whether as the single mutation or in *cis* with the D378T mutation, remain retained in the ER.

### The Double Mutant Proteins Pma1-D378T/L151F and Pma1-D378T/ΔC 409-A412, and the Single Pma1-ΔC409-A412 Form Aggregates

**Figure 9:**
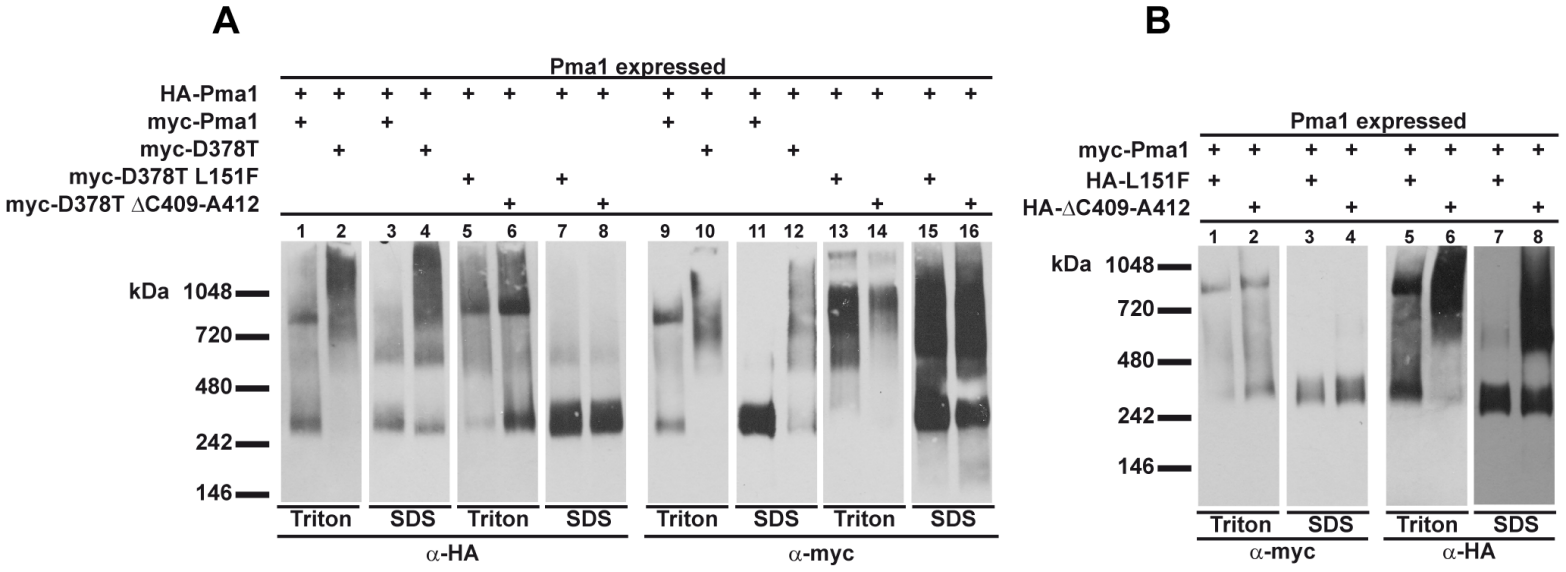
Analysis of the protein complexes formed by wild type and mutant Pma1 variants. **A)** Total membranes from yeast strains expressing HA-tagged wild type Pma1 and myc-tagged wild type Pma1 (lanes 1,3, 9 and 11), dominant lethal myc-Pma1-D378T (lanes 2, 4, 10, and 12), double mutant protein myc-Pma1-D378T/L151F (lanes 5, 7, 13, and 15) or myc-Pma1-D378T/ΔC409-A412 (lanes 6, 8, 14 and 16) were treated with 3% Triton X-100 (lanes 1, 2, 5, 6, 9, 10, 13 and 14) or 1% SDS (lanes (3, 4, 7, 8, 11, 12, 15 and 16) as described in Materials and Methods. The solubilized proteins were separated by BN-PAGE in duplicated gels and immunoblotted with anti-HA (lanes 1–8) or anti-myc (lanes 9–16) antibodies. The relative mobilities of molecular mass markers are indicated on the left. **B)** Total membranes from yeast strains expressing myc-tagged wild type Pma1 and single mutant protein HA-Pma1-L151F (lanes 1, 3, 5 and 7) or HA-Pma1-ΔC409-A412 (lanes 2, 4, 6 and 8) were treated with Triton X-100 (lanes 1, 2, 5 and 6) or 1% SDS (lanes (3, 4, 7 and 8) and the solubilized proteins separated by BN-PAGE and immunoblotted with anti-myc (lanes 1–4) or anti-HA (lanes (5–8).

We have observed by indirect immunofluorescence (see above) that the double mutant proteins Pma1-D378T/L151F and Pma1-D378T/ΔC409-A412 as well as the single mutant Pma1-ΔC409-A412 are retained in intracellular punctate structures while the accompanying wild type Pma1 reaches the plasma membrane. These results, together with previously reported data [28] showing the formation of aggregates containing mutant Pma1 proteins carrying different dominant lethal mutations, prompted us to analyze the nature of the oligomers formed by the mutant ATPases expressed from the suppressor alleles and the co-expressed wild type proteins. For this purpose, membrane proteins prepared from strains co-expressing a myc-tagged double mutant and a wild type HA-Pma1 were extracted with a non-ionic detergent, in order to preserve native structures, and subjected to blue native gel electrophoresis (BN-PAGE) [38] to examine the migration of the oligomers formed by Pma1. This method allows the preservation of the tertiary and quaternary structures of protein complexes but, nevertheless, allows the separation of the complexes according to their molecular weight. Microsomal membranes from cells co-expressing the myc-tagged double mutant alleles and the HA-tagged Pma1 were treated with Triton X-100 and analyzed by BN-PAGE and immunoblot. Membranes from cells expressing both myc- and HA-tagged wild type Pma1 or myc-Pma1-D378T and HA-Pma1 were also analyzed as control. In addition, duplicate samples of the membranes were treated with SDS and run in parallel. The results, presented in Figure 9A, showed that Triton X-100 treated membranes of wild type HA-Pma1 co-expressed with wild type myc-Pma1 presented two bands that correspond to the monomeric and oligomeric forms of Pma1 (Figure 9A, lanes 1 and 9). This behavior of Pma1, that migrates in BN-PAGE as a protein larger than its reported 100 kDa size has been previously reported [12], [28]. On the other hand, wild type HA-Pma1 showed a diffuse smear when co-expressed with the mutant myc-Pma1-D378T (Figure 9A, lanes 2 and 10). The corresponding samples treated with SDS showed predominantly the monomeric form in the case of the co-expressed wild type forms (Figure 9A, lanes 3 and 11), and in the case of HA-Pma1 co-expressed with the dominant lethal allele *PMA1*-D378T, we observed, in addition to the monomer, a smear of high molecular weight aggregates (Figure 9A, lanes 4 and 12), indicating that these forms are insoluble even in SDS. This behavior, due to the presence of oligomeric structures different from the wild type oligomer and formed when myc-Pma1-D378T is present, is attributed to the aberrant folding of the mutant protein that in turn induces misfolding of the co-expressed wild type [28]. When the myc-tagged double mutant proteins Pma1-D378T/L151F and Pma1-D378T/ΔC409-A412 were analyzed we observed high molecular weight aggregates both in Triton X-100 and SDS treated membranes (Figure 9A, lanes 13, 14, 15 and 16) very similar to those observed for myc-Pma1-D378T and the co-expressed wild type. However, wild type HA-Pma1 co-expressed with the myc-tagged double mutants behaved similar to a wild type both in Triton X-100 (Figure 9A, lanes 5 and 6) where it can be appreciated the monomeric and oligomeric bands, and in SDS (Figure 9A, lanes 7 and 8), where only the monomer was observed indicating that the presence of the suppressor mutation attenuates the formation of those high molecular weight aggregates with the accompanying wild type Pma1.

When the behavior of the single mutants co-expressed with the wild type was analyzed it was found that the wild type accompanying either one of the single mutant variants HA-Pma1-L151F or HA-Pma1-ΔC409-A412 exhibited a wild type pattern in Triton X-100 and SDS treated membranes (Figure 9B, lanes 1, 2, 3, and 4). The analysis of Pma1-ΔC409-A412 showed the existence of high molecular weight aggregates both in Triton X-100 and SDS treated membranes (Figure 9B, lanes 6 and 8) very similar to those observed for myc-Pma1-D378T and the co-expressed wild type and for the double mutant proteins Pma1-D378T/L151F and Pma1-D378T/ΔC409-A412. At a difference with these double and single mutant variants, the mutant protein Pma1-L151F behaved as a wild type protein in all conditions (Figure 9B, lanes 5 and 7). The results obtained with the single mutant proteins are consistent with those obtained by immunofluorescence and cycloheximide pulse-chase experiments, where Pma1-ΔC409-A412 was found in the punctate structures characteristic of the aggregated protein Pma1-D378T and showed increased instability, while Pma1-L151F was detected in the cell surface and was as stable as the wild type Pma1.

## Discussion

Dominant lethal mutations in the essential gene *PMA1* cause an aberrant conformation of the encoded protein that results in its retention in the ER [17], [20], [21], and degradation by the ERAD mechanism because of their recognition by the ER quality control [22], [27], [23]. It is accepted that the misfolded proteins expressed from these dominant lethal alleles, such as *PMA1*-D378T, hetero-oligomerize with the wild type enzyme and this is the cause of the co-retention of both mutant and wild type proteins [22], [28]. Here, we performed a genetic screening to isolate intragenic suppressors of the *PMA1*-D378T mutation expressed from a *GAL1* conditional promoter. We identified two suppressor mutations that restored the cells ability to grow on galactose, one of them, L151F, predicted to lie in TM2 and the other, ΔC409-A412, located in the N-domain of the protein, both affecting conserved positions of the fungal P-type ATPase protein family. We also analyzed the stability and subcellular localization of the double mutant Pma1 variants. Our results show that the second-site suppressor mutations did not significantly modified the stability of the mutant proteins, that are degraded, in a Ubc7-dependent manner, at a rate similar to that of the Pma1-D378T variant. Our results also show that neither suppressor mutation corrected the intrinsic structural defect associated with the original mutation since both double mutant proteins, Pma1-D378T/L151F and Pma1-D378T/ΔC409-A412, presented the punctate staining pattern similar to the one described for the Pma1-D378T mutant protein, indicating that the suppression mechanism does not improve the mutants capacity to exit the ER and traffic to the plasma membrane. In contrast, an allele non-specific intragenic suppressor, P536L, was found to suppress several dominant lethal *PMA1* alleles, D378N and D378E among others, but not D378T, by a mechanism involving an improved folding of the mutant protein that alleviates its ER-retention and favors its transport to the plasma membrane [43]. This mechanism of suppression was also described for second-site mutations A165V and A169I/D170N, able to suppress dominant lethal mutant *PMA1*-K474R [44]. More recently, a similar intragenic suppressor screen was performed for *YOR1*-I1084P, a mutation that induces misfolding and ER-retention of the transmembrane protein Yor1. The suppressor mutations found in that case were able to partially rescue the assembly defect caused by the primary change and restored the capacity of the protein to exit the ER [45]. Nonetheless, we did observe a change regarding the subcellular localization of the co-expressed wild type Pma1 that was now able to reach the plasma membrane, providing an explanation for the suppression of the inability to grow on galactose of the original mutant. To try to understand the structural defect that impedes normal traffic of the double mutant proteins to the plasma membrane we analyzed the assembly state of the mutant and co-expressed wild type proteins. Previous results had shown that Pma1-D378T does not form a normal oligomer with wild type Pma1, but instead the misfolded protein interferes with the folding of the wild type leading to the formation of aggregates that are insoluble even in SDS as revealed by BN-PAGE [28]. When this type of analysis was performed with the double mutant proteins Pma1-D378T/L151F and Pma1-D378T/ΔC409-A412, we observed that the co-expressed wild type recovered almost completely the wild type pattern, as shown by the presence of the monomeric and oligomeric bands characteristic of Pma1 from Triton X-100 membranes. This rescue of the co-expressed wild type protein is consistent with its detection at the plasma membrane, and contrasts with the behavior of the accompanying double mutant proteins that form insoluble aggregates as those formed by Pma1-D378T and are unable to reach the cellular surface. In the light of these and previous results it can be concluded that at least two different suppression mechanisms exist for dominant lethality. One of them improves the folding of the revertant protein and favors its transport to the plasma membrane, as exemplified by mutations P536L, A165V and A169I/D170N [43], [44]. The other mechanism, shown by the L151F and ΔC409-A412 mutations described here, does not rescue the folding and trafficking of the aberrant dominant lethal protein but disrupts the formation of oligomers between the mutant and wild type enzymes allowing wild type Pma1 to exit the ER and traffic to the plasma membrane, while the revertant enzyme remains aggregated in the ER. A similar way of suppression is used by the previously described Trp859stop mutation [28]. Thus, the suppressor mutations found for the D378T mutation in two independent genetic screenings share the suppression mechanism.

The single missense mutation L151F rendered a Pma1 variant whose stability and final localization in the plasma membrane are very similar to that of the wild type protein. When analyzed by BN-PAGE, it showed the monomeric and oligomeric wild type forms in TritonX-100 treated membranes, as opposed to Pma1-ΔC409-A412 that presented high molecular weight aggregates insoluble in SDS as the double mutants did.

Leu151 lies in the middle of the predicted segment TM2 within the primary sequence: WVDFGVICGL**L**MLNAGVGFV. A number of mutations in TM2 were recovered as second-site revertants of different primary mutations, for instance mutations I147M and C148S were found to suppress S368F, a mutation located in close vicinity to D378T, (see [18], for a review). These and other genetic data obtained by site-directed mutagenesis [46] have led to the notion that TM1 and TM2 form a conformationally sensitive hairpin structure. However a silent change in these helices is not unprecedented since a mutation F120W, in TM1, gave a fully functional enzyme [46]. On the other hand, when this change is present in the D378T allele the resultant double mutant protein is unable to oligomerize with the wild type Pma1, as shown by the BN-PAGE analysis, pointing to a possible role for TM2 in the intramembrane assembly process between monomers. Cys409 is in the central hydrophilic domain of the protein, located between the phosphorylation site, Asp387, and the N-domain. In a study dedicated to the role of cysteine residues in the structure and function of Pma1 [41] it was found that a substitution C409A enhanced the reactivity of another cysteine residue, Cys532, towards N-Ethylmaleimide. The C409A change also increased the sensitivity to fluorescein 5′-isothiocyanate, an inhibitor of ATPase known to react quite specifically with Lys474. This work suggests that a C409A substitution leads to a conformational change in this region of the protein. More recently, the role of the large hydrophilic domain in Pma1 oligomerization has been addressed by studying the association state of this domain expressed in *E.coli* [47], reaching the conclusion that the phosphorylation domain plays a fundamental role in the dimerization process. Taking these data into account we propose that the 4 amino acids deletion in this region present in the proteins Pma1-D378T/ΔC409-A412 and Pma1-ΔC409-A412, alters the conformation of the protein in such a way that disrupts the interaction with the co-expressed wild type Pma1.

The two second-site mutations identified in this work have the common effect of altering the conformation of the mutant proteins and interfering with the oligomerization process that precedes ER export of Pma1. The location of these mutations suggests the involvement of TM2 and P-domain in the assembly of the Pma1 oligomers.

## Supporting Information

Figure S1 **Suppression of dominant lethal mutations** ***PMA1*** **-D560N and** ***PMA1*** **-R695L.** Drop test for growth on galactose of yeast strains carrying the indicated *PMA1* alleles. Yeast strain BY4741 was transformed with centromeric plasmid pRS316 carrying *GAL1*-driven *PMA1*, *PMA1*-D560N, *PMA1*-R695L, *pma1*-D560N/L151F, *pma1*-D560N/ΔC409-A412, *pma1*-R695L/L151F or *pma1*-R695L/ΔC409-A412 alleles. The different transformants were grown in SR medium for 24 h, suspended in water to an OD_660_ = 0.1 and 5 µl were dropped on SD and SG agar plates. Identical results were obtained with three independent transformants. To create the double mutants containing L151F and either D560N or R695L mutations, a 3,4 kb *BstE*II-*Hind*III DNA fragment containing the dominant lethal mutations, was excised from the *PMA1*-D560N or *PMA1*-R695L genes and cloned into the same sites of pRS316-*GAL1*-HA-*pma1*-L151F. Site-directed mutagenesis was used to introduce the D560N and R695L mutations respectively in a 4.3 kb *Xho*I-*Hind*III fragment containing the HA-tagged *pma1-*ΔC409-A412 gene subcloned into pSK vector. The new *PMA1* mutants were cloned into pRS316 in which *GAL1* promoter had been previously introduced.
(TIF)

Figure S2 **Stability of the Pma1 mutants in** ***ubc7*** **background.** Yeast strain BY4741 *MAT*a *his*3Δ1 *leu2*Δ0 *met15*Δ0 *ura3*Δ0 YMR022w::kanMX4 carrying a disruption in *UBC7* was purchased from EUROSCARF and transformed with centromeric plasmid pRS315 carrying *GAL1*-driven myc-*PMA1*-D378T or double mutant alleles myc-*pma1*-D378T/L151F and myc-*pma1*-D378T/ΔC409-A412. The transformants were grown in SR medium and derepressed in galactose containing medium for 4 h to induce the expression of the different *PMA1* alleles. After addition of cycloheximide, samples were taken at the indicated times and total yeast membranes prepared and analyzed by Western blot with anti-myc antibodies. A representative blot of two cycloheximide-chase experiments is shown. Densitometric analysis of the Western blots is also shown. Remaining Pma1 at different times was referred to the amount before adding cycloheximide (t = 0) and the average of the two experiments is shown. Dashed lines representing the results of the same experiment performed in wild type background (Figure 4) are included here for comparison. Pma1-D378T (circles), Pma1-D378T/L151F (squares), Pma1-D378T/ΔC409-A412 (triangles).
(TIF)
