# Supplementary figures and images for: Characterization of Two Second-Site Mutations Preventing Wild Type Protein Aggregation Caused by a Dominant Negative PMA1 Mutant
Source: PLoS One. 2013 Jun 25;8(6):e67080. doi: 10.1371/journal.pone.0067080 (PMC3692421; doi:10.1371/journal.pone.0067080)

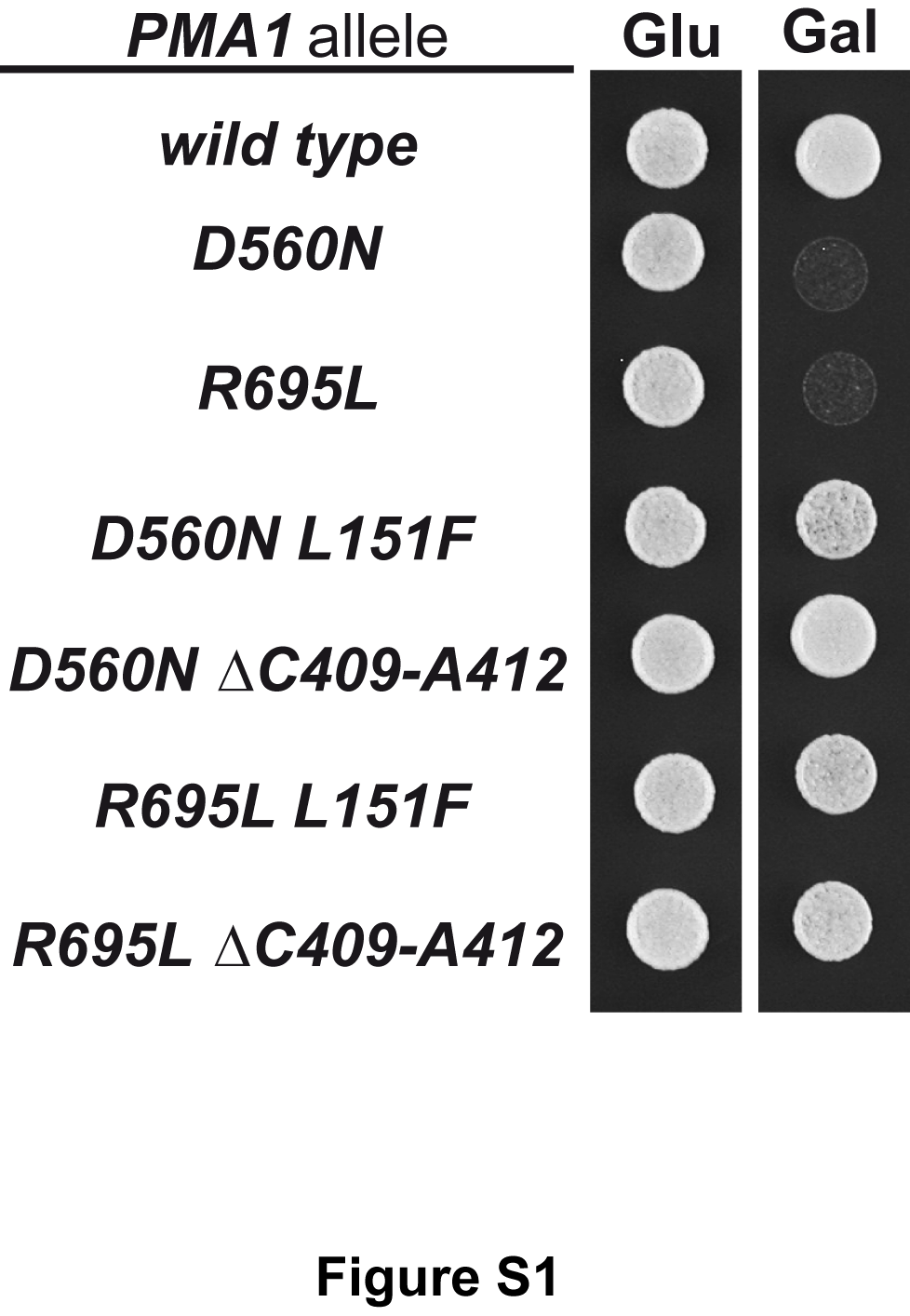

Supplement: Figure S1 — Suppression of dominant lethal mutations PMA1 -D560N and PMA1 -R695L. Drop test for growth on galactose of yeast strains carrying the indicated PMA1 alleles. Yeast strain BY4741 was transformed with centromeric plasmid pRS316 carrying GAL1-driven PMA1, PMA1-D560N, PMA1-R695L, pma1-D560N/L151F, pma1-D560N/ΔC409-A412, pma1-R695L/L151F or pma1-R695L/ΔC409-A412 alleles. The different transformants were grown in SR medium for 24 h, suspended in water to an OD660 = 0.1 and 5 µl were dropped on SD and SG agar plates. Identical results were obtained with three independent transformants. To create the double mutants containing L151F and either D560N or R695L mutations, a 3,4 kb BstEII-HindIII DNA fragment containing the dominant lethal mutations, was excised from the PMA1-D560N or PMA1-R695L genes and cloned into the same sites of pRS316-GAL1-HA-pma1-L151F. Site-directed mutagenesis was used to introduce the D560N and R695L mutations respectively in a 4.3 kb XhoI-HindIII fragment containing the HA-tagged pma1-ΔC409-A412 gene subcloned into pSK vector. The new PMA1 mutants were cloned into pRS316 in which GAL1 promoter had been previously introduced. (TIF) [file pone.0067080.s001.tif]

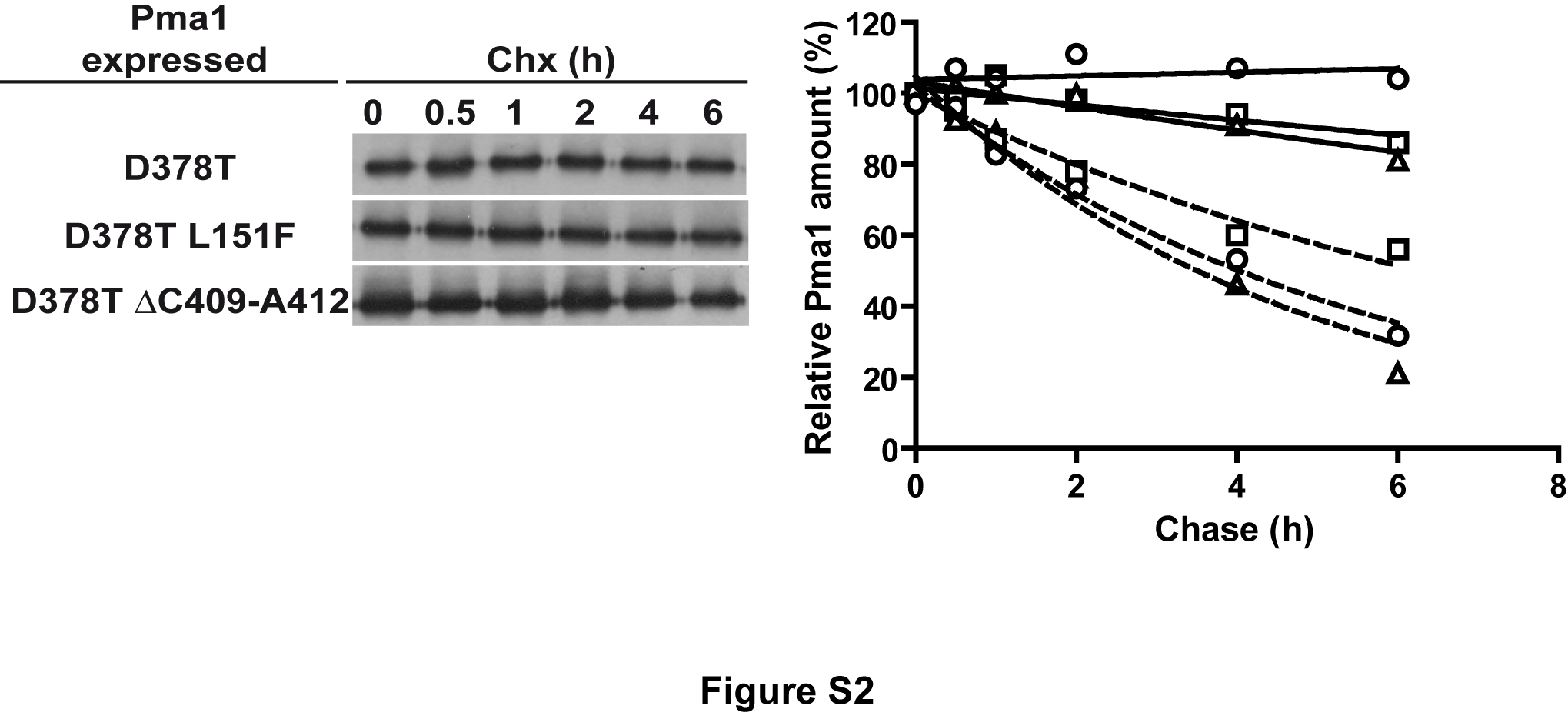

Supplement: Figure S2 — Stability of the Pma1 mutants in ubc7 background. Yeast strain BY4741 MATa his3Δ1 leu2Δ0 met15Δ0 ura3Δ0 YMR022w::kanMX4 carrying a disruption in UBC7 was purchased from EUROSCARF and transformed with centromeric plasmid pRS315 carrying GAL1-driven myc-PMA1-D378T or double mutant alleles myc-pma1-D378T/L151F and myc-pma1-D378T/ΔC409-A412. The transformants were grown in SR medium and derepressed in galactose containing medium for 4 h to induce the expression of the different PMA1 alleles. After addition of cycloheximide, samples were taken at the indicated times and total yeast membranes prepared and analyzed by Western blot with anti-myc antibodies. A representative blot of two cycloheximide-chase experiments is shown. Densitometric analysis of the Western blots is also shown. Remaining Pma1 at different times was referred to the amount before adding cycloheximide (t = 0) and the average of the two experiments is shown. Dashed lines representing the results of the same experiment performed in wild type background (Figure 4) are included here for comparison. Pma1-D378T (circles), Pma1-D378T/L151F (squares), Pma1-D378T/ΔC409-A412 (triangles). (TIF) [file pone.0067080.s002.tif]
